# Phase I trial of a multi-epitope-pulsed dendritic cell vaccine for patients with newly diagnosed glioblastoma

**DOI:** 10.1007/s00262-012-1319-0

**Published:** 2012-07-31

**Authors:** Surasak Phuphanich, Christopher J. Wheeler, Jeremy D. Rudnick, Mia Mazer, HongQian Wang, Miriam A. Nuño, Jaime E. Richardson, Xuemo Fan, Jianfei Ji, Ray M. Chu, James G. Bender, Elma S. Hawkins, Chirag G. Patil, Keith L. Black, John S. Yu

**Affiliations:** 1 Neuro-Oncology Program, Department of Neurosurgery and Neurology, Cedars-Sinai Medical Center, 8631 W. 3rd Street Suite 410 E, Los Angeles, CA 90048 USA; 5 Neuro-Oncology Program, Department of Neurosurgery, Cedars-Sinai Medical Center, 8631 W. 3rd Street Suite 800 E, Los Angeles, CA 90048 USA; 2 ImmunoCellular Therapeutics Ltd., Woodland Hills, CA USA; 3 Department of Neurosurgery, Cedars-Sinai Medical Center, 110 N. George Burns Road, Davis 2097, Los Angeles, CA 90048 USA; 4 Department of Pathology, Cedars-Sinai Medical Center, 8700 Beverly Blvd. room 8725, Los Angeles, CA 90048 USA

**Keywords:** Dendritic cell immunotherapy, Cancer stem cells, Cancer vaccine, CTL, Epitopes, Glioblastoma

## Abstract

**Background:**

This study evaluated the safety and immune responses to an autologous dendritic cell vaccine pulsed with class I peptides from tumor-associated antigens (TAA) expressed on gliomas and overexpressed in their cancer stem cell population (ICT-107).

**Methods:**

TAA epitopes included HER2, TRP-2, gp100, MAGE-1, IL13Rα2, and AIM-2. HLA-A1- and/or HLA-A2-positive patients with glioblastoma (GBM) were eligible. Mononuclear cells from leukapheresis were differentiated into dendritic cells, pulsed with TAA peptides, and administered intradermally three times at two-week intervals.

**Results:**

Twenty-one patients were enrolled with 17 newly diagnosed (ND-GBM) and three recurrent GBM patients and one brainstem glioma. Immune response data on 15 newly diagnosed patients showed 33 % responders. TAA expression by qRT-PCR from fresh-frozen tumor samples showed all patient tumors expressed at least three TAA, with 75 % expressing all six. Correlations of increased PFS and OS with quantitative expression of MAGE1 and AIM-2 were observed, and a trend for longer survival was observed with gp100 and HER2 antigens. Target antigens gp100, HER1, and IL13Rα2 were downregulated in recurrent tumors from 4 HLA-A2+ patients. A decrease in or absence of CD133 expression was seen in five patients who underwent a second resection. At a median follow-up of 40.1 months, six of 16 ND-GBM patients showed no evidence of tumor recurrence. Median PFS in newly diagnosed patients was 16.9 months, and median OS was 38.4 months.

**Conclusions:**

Expression of four ICT-107 targeted antigens in the pre-vaccine tumors correlated with prolonged overall survival and PFS in ND-GBM patients. The goal of targeting tumor antigens highly expressed on glioblastoma cancer stem cells is supported by the observation of decreased or absent CD133 expression in the recurrent areas of gadolinium-enhanced tumors.

## Introduction

Glioblastoma multiforme (GBM) is the most common and malignant primary brain tumor [1]. Immunotherapy with dendritic cells (DCs) offers the potential for high tumor-specific toxicity and sustained tumoricidal activity [2–4]. DCs are the most potent antigen-presenting cells and can be derived ex vivo from blood monocytes using GM-CSF and IL-4 [5]. DC immunotherapy has been studied in a wide variety of cancers including patients with glioma [6–8]. Our previous immunotherapy trials have demonstrated induction of tumor-specific immune responses [2, 6, 9] that correlated with clinical outcomes [2].

Cancer stem cells (CSCs) have recently been identified in human brain tumors [10, 11]. This minority cell population demonstrates potent tumorigenicity in immunodeficient animals and shares properties with their normal stem cell counterparts, namely the expression of CD133, the properties of self-renewal, and differentiation into comprehensive neuronal and glial lineages [12]. CD133+ CSCs in glioma are more radioresistant [13] and chemoresistant [14] and have been correlated with poor clinical outcome [15], and studies of recurrent tumors have shown increased expression of CD133 [14, 16]. Vaccine studies in mice using lysates from CSC showed superior protective immunity compared with lysates from whole tumor [17, 18]. We thus pursued a clinical strategy of DC vaccination targeting antigens overexpressed on CSCs along with other known tumor-specific antigens.

Tumor-associated antigens have been identified that are expressed on GBM cells [19, 20] and overexpressed on the CSC [17]. These include HER2/neu [19, 21], TRP-2 [19, 20, 22], and AIM-2 [19, 23]. Other known tumor antigens have been shown to be expressed on GBM cells including gp100 [19–21], MAGE1 [19, 21], and IL13Ra2 [19, 20, 24]. ICT-107 is an autologous vaccine consisting of patient DC pulsed with six synthetic class I peptides from AIM-2, MAGE1, TRP-2, gp100, HER2/neu, and IL-13Rα2. We report here the safety and biological responses of ICT-107 in patients with gliomas.

## Patients and methods

### Study design

**Fig. 1:**
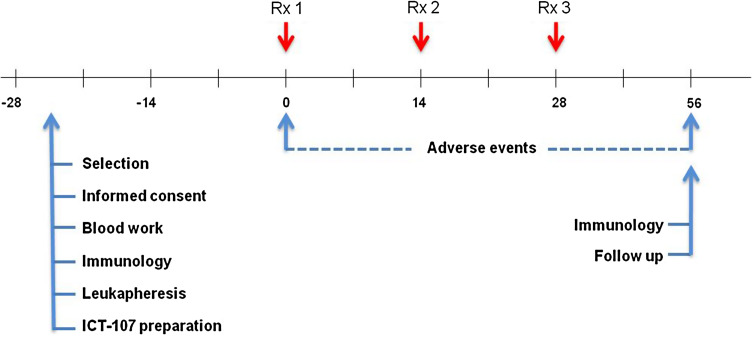
Diagram of treatment schedule and events

This was an open-label, single-institution, single-arm, phase I study with the primary endpoint to evaluate the immunogenicity of ICT-107 and secondary endpoints of safety and efficacy in patients with glioblastoma or brainstem glioma. ND-GBM patients with surgery, who had no imaging of tumor progression after a standard treatment with concurrent temozolomide (TMZ) and radiation therapy, were eligible for vaccine therapy, and recurrent GBM patients with a gross total resection were also eligible. After explanation of the protocol, written informed consent was obtained from patients before screening; the vaccine was then prepared and administered intradermally in the axilla region every 2 weeks for three consecutive doses after the completion of radiation or surgery. A diagram of the treatment schedule is shown in Fig. 1. Post-vaccine, newly diagnosed patients received maintenance temozolomide chemotherapy following the last dose of vaccine and recurrent patients received chemotherapy with or without bevacizumab. Patients were monitored once a month by neurological examination and every 2 months by magnetic resonance imaging. Progressive disease was defined by MacDonald criteria. This protocol was allowed by the US FDA and the local institution review board at Cedars-Sinai Medical Center.

### Patient selection

Patients with newly diagnosed or recurrent GBM with a gross total resection (>95 %) or brainstem glioma were HLA-A1 or HLA-A2 positive (HLA typing done by low resolution PCR-SSOP). Inclusion criteria included the presence of at least one of the vaccine antigens on the patient’s tumor, a Karnofsky score of at least 60 %, glucocorticoid therapy with dexamethasone ≤4 mg/day, and normal baseline hematological parameters. Exclusion criteria included pregnancy; severe pulmonary, cardiac, or other systemic disease associated with an unacceptable anesthetic or operative risk; presence of an acute infection requiring active treatment; history of an autoimmune disorder or allergy to gentamicin; or prior history of other malignancies, excluding basal cell carcinoma and benign tumors. The extent of surgical resection was defined as either complete resection, with no linear gadolinium enhancement on T1 MRI image or sub-total resection in which minimal residual nodules were observed.

### Tumor characterization

Tumor samples collected from surgery were dissociated into single-cell suspensions with trypsin–EDTA (Invitrogen, Carlsbad, CA) for 30 min and frozen at −80 °C. In some samples, formalin-fixed paraffin-embedded (FFPE) samples were evaluated. RT-PCR method and primers used to quantitate expression of mRNA for MAGE1, gp100, HER2, AIM-2, TRP-2, and CD133 were previously described [14, 17, 21–23]. IL13Ra2 primers were from Qiagen, Valencia, CA. Data analysis was done by comparison with the GAPDH control using the 2^−ΔΔ CT^ method [25].

FFPE tumor samples were evaluated for MGMT methylation by MDxHealth (Liege, Belgium) using RT-PCR as previously described [26] and PTEN immunohistochemistry with PTEN antibody (Clone 6H2.1, Cascade Biosciences). Immunoreactivity was detected and visualized with either an Ultraview DAB or a Bond Refine DAB system and reviewed by at least one board-certified neuropathologist.

### Preparation of autologous vaccine

Monocytes were prepared from leukapheresis products by adherence for 2 h at 37 °C, and adherent cells were cultured for 5 days in RPMI 1640 with 10 % autologous serum supplemented with recombinant human granulocyte–macrophage colony-stimulating factor (Berlex) (800 units/ml) and interleukin-4 (R&D systems) (500 units/ml). On Day 5, 50 ng/ml tumor necrosis factor-α (R&D systems) was added for 3–4 days.

Peptides included HLA-A1 restricted, MAGE1(161) EADPTGHSY, AIM-2(14) RSDSGQQARY, and HLA-A2 restricted, TRP-2(180) SVYDFFVWL, gp100(210M) IMDQVPFSV, HER2(773) VMAGVGSPYV and IL13Rα2(345) WLPFGFILI (Clinalfa, Läufelfingen, Switzerland). Cells from Day 8–9 cultures were cultured at 10^6^/ml with peptides (10 μg/ml per antigen) at 37°/5 % CO_2_, 16–20 h. Pulsed cells were resuspended at 1 × 10^7 ^cells/ml in 10 %DMSO/90 % autologous serum and 1.1 ml, dispensed into 1.8 ml Nunc cryo tubes (Roskilde, Denmark), frozen at 1 °C/min, and stored in the vapor phase of liquid nitrogen.

On culture Day 2, a media aliquot was tested for sterility. A gram stain, sterile culture, mycoplasma, and LAL endotoxin assay were performed on the final product before administration.

### Vaccine administration

The frozen dose was thawed rapidly in a 37 °C water bath, transferred into a 1 ml tuberculin syringe, and administered intradermally at multiple sites in the axillary region. Patients were monitored for 2 h post-immunization for any adverse effects. Patients received pretreatment with 50 mg of diphenhydramine and 650 mg of acetaminophen as needed.

### Immune response methods

Pre- and post-vaccination (7 days prior to vaccination, and 56 days later: 5 weeks after administration of the third and last vaccine), PBMCs were thawed and co-cultured with a mixture of all immunizing peptides (10 μg/ml) in stimulating medium (10 % FBS/RPMI+ anti-CD28/CD49d) at 37° C for 7 days.

On Day 7, cultures were restimulated with peptides. Brefeldin-A and monensin were added after 1 h, and the cells were cultured five more hours. Washed cells were stained with surface markers (CD8, clone OKT8; CD3, clone OKT3; CD107a, clone eBioH4A3, IFNγ, clone 4S.B3; TNFα, MAb11; all eBioscience), resuspended in 1X PermiFlow (Invirion) and stored at room temperature overnight. Intracellular staining for IFNγ and TNFα was performed and the CD8^+^INFγ^+^ population quantified within both CD8^hi^ and CD8^lo^ subpopulations, as recommended by Cancer Immunotherapy Consortium Immune Monitoring Panels[27], using identical forward and side scatter gating in all samples (Fig. 6). A proportion of vaccine-enhanced cytokine^+^ CD8^+^ cells [(Ag stimulated/unstimulated CD8^+^ post-vaccine) ÷ (Ag stimulated/unstimulated CD8^+^ pre-vaccine)] were used to assess immune responsiveness. CD107a staining closely paralleled that of IFNγ and TNFα (not shown) and correlated significantly with IFNγ responses (*r* = 0.65 for all cytokine^+^, and 0.932 for CD8^hi^ gates; *p* = 0.01 and 0.001, respectively). Patients with a 1.5-fold higher post-vaccine than with pre-vaccine score were considered responders. This protocol reproduced immune response results of previous autologous DC stimulation assays analyzed by quantitative PCR for IFNγ production at Day 56 [2], without requiring high volume blood collections for DC differentiation (not shown).

### Statistical analysis

Continuous variables were compared with Student’s *t* test and categorical variables compared using Fisher’s exact test. Probability of survival was determined with SAS software by the Kaplan–Meier method, using two-tailed Mann–Whitney log-rank test exclusively to compare groups. Pearson’s correlation coefficients (*r* values) were calculated in Excel software. Standard errors (±) of the mean were provided whenever appropriate. Significant observations were considered for *p* < 0.05.

## Results

### Patient characteristics

Twenty-one patients who were HLA-A1 and/or HLA-A2 positive, 16 males and 5 females, were enrolled between May 2007 and January 2010 (Table 1). There were 17 ND-GBM patients (16 evaluable; one patient did not receive treatment), three patients with recurrent GBM, and one with brainstem glioma (Table 1). A total of 62 dendritic cell vaccinations were administered. In general, the vaccinations were well tolerated with only grade 1 or 2 adverse events (Table 2). The median age was 52 years (range 26–79 years), and the median Karnofsky score was 90 (range 70–100). ND-GBM patients that subsequently progressed were treated with a variety of additional therapies including bevacizumab, CPT-11, dose-dense temozolomide and cediranib.

**Table 1:** Baseline characteristics of recurrent/newly diagnosed glioblastoma patients

	Recurrent and newly diagnosed	Newly diagnosed
	*N* = 19	*N* = 16
Gender		
Male, *N* ( %)	15 (78.9)	12 (75.0)
Age in years		
Mean (SD)	51.8	55.3 (10.7)
Median	52	54.5
Range	26–79	34–79
Karnofsky status^a^		
Mean (SD)	87 (9)	87 (10.1)
Median	90	90
Range	60–100	60–100
HLA status, *N* (%)		
A1+	6	5
A2+	12	10
A1+/A2+	1	1
Prior therapy, *N* (%)		
Surgery	19	16
Radiotherapy	19	16
Temozolomide	19	16
Avastin	2	
Gliadel	4	2
Extent of surgery, *N* (%)		
Sub-total resection	4	4
Complete resection	15	12
First surgery to vaccine time (months)		
Mean (SD)	7.32 (5.69)	5.49 (2.52)
Median	5.19	4.52
Range	2.96–23.01	2.96–12.39
Corticosteroid therapy (%)	26.3	18.7

^a^Score collected at screening

**Table 2:** Attributable adverse events

Event	Number of patients (*N* = 11)			Number of events (*N* = 12)		
	Grade 1 *N* (%)	Grade 2 *N* (%)	Total grade 1 and 2	Grade 1 *N* (%)	Grade 2 *N* (%)	Total grade 1 and 2
Diarrhea	–	1 (9)	1 (9)	–	1 (8)	1 (8)
Fatigue	3 (27)	–	3 (27)	4 (33)	–	4 (33)
Flushing	1 (9)	–	1 (9)	1 (8)	–	1 (8)
Pruritus	2 (18)	–	2 (18)	2 (17)	–	2 (17)
Rash	2 (18)	–	2 (18)	2 (17)	–	2 (17)
Redness on skin	1 (9)	–	1 (9)	1 (8)	–	1 (8)
Vomiting	–	1 (9)	1 (9)	–	1 (8)	1 (8)

### Tumor characterization

Pathological examination of tumors showed variable morphologic features of glioblastoma multiforme (GBM). MGMT methylation by PCR and PTEN expression by immunohistochemistry were performed on the majority of cases. (Table 3). MGMT methylation was observed in 60 % (6 of 10) of the newly diagnosed patients and in 47 % (7 of 15) of all the patients with evaluable results. Six of the newly diagnosed patients did not have samples available for testing or had invalid results. PTEN expression by immunohistochemistry was retained in 8 of 15 (53 %) newly diagnosed patients evaluated.

**Table 3:** Characterization of tumor for newly diagnosed glioblastoma patients

Patient ID	Ag expression on tumor (peptide HLA restriction)							Biomarker characterization	
	HLA type	AIM2 (A1)	gp100 (A1)	MAGE (A2)	TRP-2 (A2)	HER2 (A2)	ILRa2 (A2)	MGMT	PTEN
2	A2+	+	+	+	+	+	+	UnMet	RETAINED
4	A1+	+	+	+	+	+	+	UnMet	N/A
6	A2+	+	+	Wk	+	+	+	Met	RETAINED
7	A2+	+	+	+	+	+	+	Met	LOSS
9	A2+	+	+	−	+	−	+	Met	RETAINED
10	A2+	+	+	Wk	+	+	+	INV	RETAINED
11	A2+	+	+	+	+	+	+	UnMet	LOSS
12	A2+	+	+	Wk	+	+	+	UnMet	RETAINED
13	A1+	+	Wk	Wk	+	+	+	NT	LOSS
14	A1+	+	+	−	+	+	+	NT	LOSS
15	A1+	+	−	+	+	+	+	INV	RETAINED
16	A1 + A2+	+	+	Wk	+	+	+	Met	LOSS
17	A2+	+	−	Wk	−	+	−	NT	LOSS
18	A1+	+	Wk	Wk	+	+	+	NT	LOSS
19	A2+	+	+	Wk	+	+	+	Met	RETAINED
20	A2+	+	+	Wk	+	+	+	Met	RETAINED

Positive (+), negative (−), weak (Wk)

*UnMet* unmethylated, *Met* methylated, *INV* invalid results, *NT* not tested

Expression of tumor-associated antigens using PCR measurement of mRNA was assessed on fresh-frozen samples from patient tumors. Previous studies have demonstrated the protein expression of these antigens on primary tumors and GBM cell lines [19–23]. All patient tumors expressed at least three of the antigens (Table 3). Fourteen (74 %) tumors expressed all six antigens, two (11 %) expressed five antigens, two (11 %) expressed four antigens, and one tumor (5 %) expressed three antigens.

**Fig. 2:**
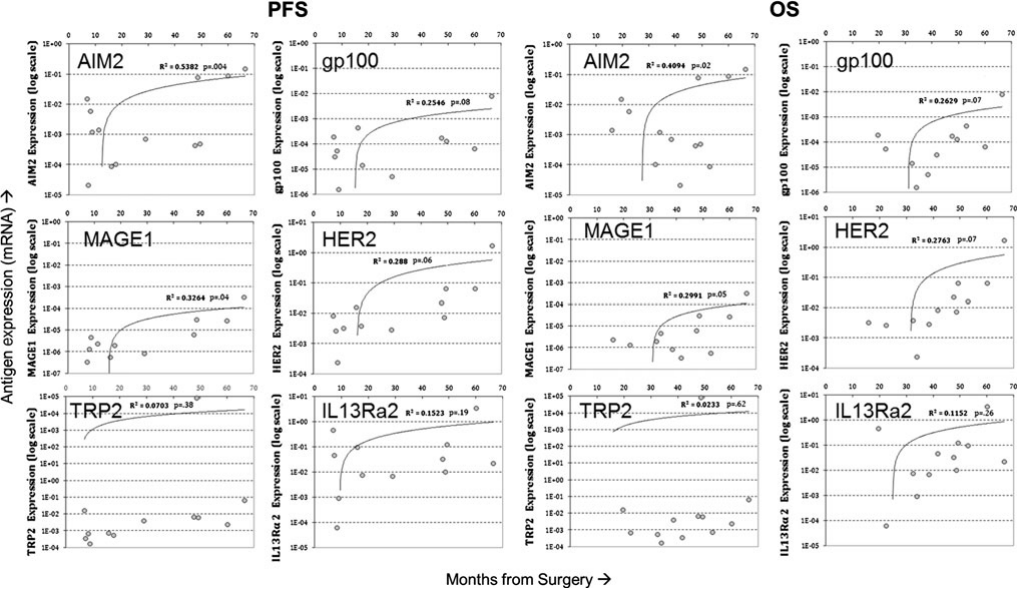
**a** Correlation of quantitative antigen expression on primary tumor from ND patients with progression-free survival (*n* = 13). Logarithmic plots of antigen expression determined by qRT-PCR (see “Patients and methods”) showed correlations of increasing antigen expression with longer progression-free survival times (PFS). Antigen expression was measured from fresh-frozen samples and calculated relative to GAPDH. **b** Correlation of quantitative antigen expression on primary tumor from ND patients with overall survival (*n* = 13). Logarithmic plots of antigen expression determined by qRTPCR (see “Patients and methods”) showed correlations of increasing antigen expression with longer overall survival time (OS)

**Fig. 3:**
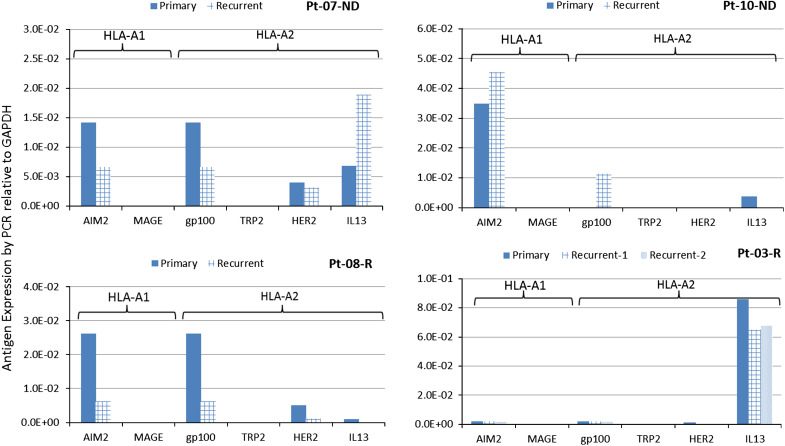
Downregulation of target antigens in recurrent tumors from HLA-A2+ patients. Antigen expression was from FFPE samples and calculated relative to GADPH. Significant downregulation post-vaccine of A2 epitopes gp100, HER2, and IL13Rα2 relative to upregulations was observed (*p* = 0.023, Fisher’s exact test). Downregulation of the HLA-A1 antigen, AIM2, was not significant (*p* = 0.21) in these patients

Quantitative expression of the TAA targeted by the vaccine was analyzed in 13 ND-GBM patients for whom these data were available. In this analysis, expression of AIM-2 and MAGE1 was shown to be significantly correlated with PFS and OS (Fig. 2) and expression of HER2 and gp100 showed a trend toward longer PFS and OS. Significant correlations were not observed with IL13Rα2 and TRP-2. FFPE samples from second surgeries in five patients were evaluated and compared with samples from primary tumors. Studies comparing matching FFPE and fresh-frozen samples with PCR measurement of antigen expression (not shown) showed a lower sensitivity of detection using FFPE samples resulting in samples that were negative for expression. Figure 3 shows the expression of target antigens in primary and recurrent tumors from four HLA-A2 patients. Significant post-vaccine downregulation (*p* = 0.023 by Fisher’s exact test) of the A2 epitopes relative to upregulation was observed in three patients where responses were positive. For comparison, downregulation of the HLA-A1 target antigen AIM2 was not significant in these patient tumors.

**Fig. 4:**
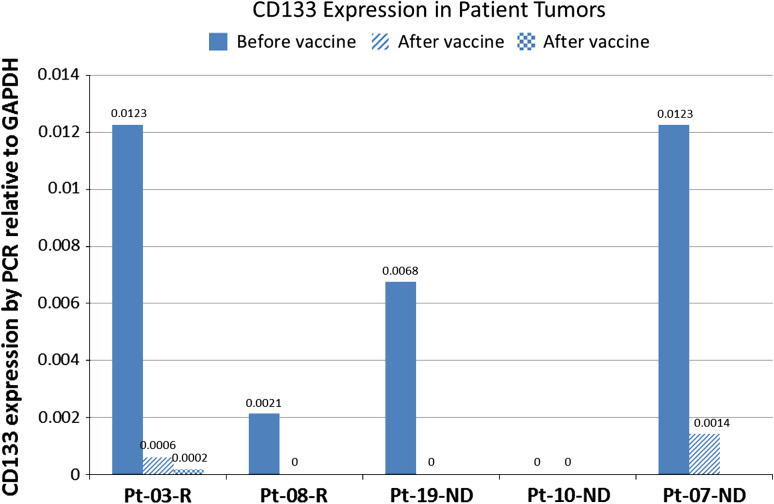
CD133 expression by RT-PCR in primary tumor and samples from subsequent surgeries from newly diagnosed and recurrent patients. Expression is from FFPE samples and calculated relative to GADPH as described in “Patients and methods”. The sample for Patient 19 was negative for tumor

CD133, a non-targeted antigen expressed on the stem cell fraction in gliomas and reported to be increased in recurrent tumors [14, 16], was evaluated in FFPE samples from primary and recurrent tumors. In recurrent patients, CD133 expression in the recurrent tumor decreased by two logs in one patient (#03) and became negative in the other (#08) (Fig. 4). In newly diagnosed patients, one patient (#10) was negative for CD133 in both the primary and recurrent tumor, one patient (#09) showed a one-log decrease in CD133 expression, and the third patient (#19) was negative for CD133 in the second surgical sample. This surgery performed on patient #19 showed no evidence of tumor, and this patient was considered not to have progressed.

### Survival

**Fig. 5:**
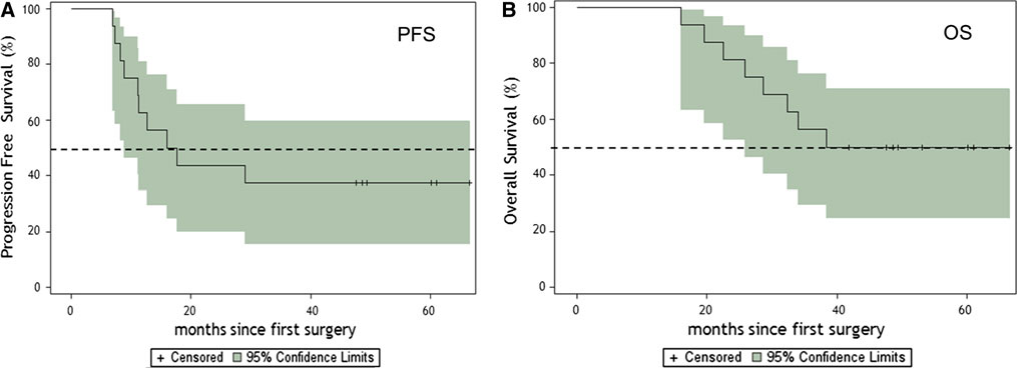
**a** Kaplan–Meier estimates of progression-free survival for newly diagnosed patients (*n* = 16). *Dotted lines* illustrate the 95 % confidence intervals, and *hash marks* denote censored patients. **b** Kaplan–Meier estimates of overall survival for newly diagnosed patients (*n* = 16). *Dotted lines* illustrate the 95 % confidence intervals, and *hash marks* denote censored patients

For survival analysis, only the 16 newly diagnosed adult GBM patients who received treatment were considered. No patient was lost to follow-up. A summary of demographics, survival, immune response, and KPS on individual newly diagnosed patients is presented in Table 4. The progression-free survival (PFS) and overall survival rates (OS) are presented in Table 5. The PFS time was 16.9 months, and a two-year PFS rate was 43.8 % (95 %CI, 19.8–66.0). The three-year overall survival rate was 55.6 % (95 %CI, 28.6–75.9). The median OS was 38.4 months with eight of 16 patients alive. Six patients showed no tumor recurrence at 49 to 66 months. The Kaplan–Meier probability curves of PFS and OS are shown in Fig. 5a and b, respectively. No difference in survival was observed between HLA-A2+ and HLA-A1+ patients.

**Table 4:** Demographics and survival and immune response for newly diagnosed glioblastoma patients

Patient ID	Age	Sex	Site	Extent of resection	Time to progression (months)	Survived time (months)	Immune response^b^ IFNγ^hi^, IFNγ^lo^	Karnofsky score^d^
2	64	M	R temporal parietal	Complete	11.15	28.60	0.76, 0.65	100
4	46	M	L frontal	Complete	60.95^a^	60.95^a^	**1.74**, 1.15	90
6	56	F	L temporal	Complete	66.51^a^	66.51^a^	1.03, 0.96	100
7	61	F	R frontal	Complete	60.10^a^	60.10^a^	**3.58** ^c^	90
9	51	M	R anterior temporal	Complete	6.87	19.53	0.67, 0.95	90
10	47	M	L frontoparietal	Complete	15.98	53.03^a^	1.49, 1.00	90
11	53	F	R temporal	Sub-total	12.62	25.91	0.61, 0.74	90
12	65	M	R temporal	Complete	8.25	22.55	0.43, 0.49	90
13	60	M	L temporal	Complete	29.0	38.37	**2.19**, 0.53	90
14	44	M	R temporoparietal	Complete	49.38*	49.38*	0.46, **1.73**	80
15	34	M	R parietal	Complete	48.66^a^	48.66^a^	0.43, 0.82	90
16	63	M	L parietal	Sub-total	17.72	32.42	1.07, 1.18	60
17	79	M	R frontal	Sub-total	11.28	15.98	0.62, **1.83**	70
18	52	F	Bifrontal	Complete	8.88	33.99	1.21, 1.01	90
19	48	M	L Frontal	Complete	47.64^a^	47.64^a^	0.56, 0.91	80
20	62	M	L Temporal	Sub-total	7.27	41.82^a^	NT	90

^a^Progression/mortality has not been observed for these patients

^b^IFNγ^hi^ = IFNγ^+^ in CD8^hi^ gate; IFNγ^lo^ = IFNγ^+^ in CD8^lo^ gate

^c^Fresh CD8^+^ cells, no in vitro stimulation

^d^At screening

Bold values indicate positive immune response greater than 1.5

**Table 5:** Progression-free and overall survival in months for newly diagnosed glioblastoma patients

Time in months	% Survival (95 % CI)
Progression-free survival	
6	100
12	62.5 (34.9, 81.1)
18	43.8 (19.8, 66.0)
24	43.8 (19.8, 66.0)
Median	16.9 (8.9, 49.8)
Overall survival	
6	100
12	100
18	93.7 (63.2, 99.1)
24	80.3 (58.6, 96.7)
36	55.6 (28.6, 75.9)
Median	38.4 (25.9, 40.7)

*CI* confidence interval

### Development of systemic type I cytokine responses and correlation with survival

**Fig. 6:**
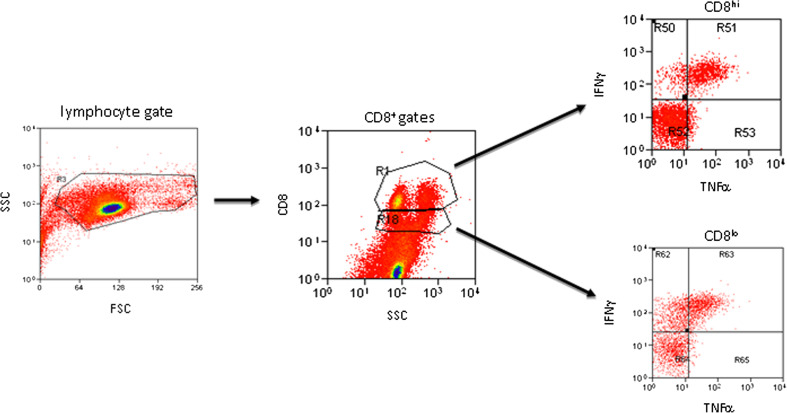
Gating strategy for intracellular cytokine analysis in CD8^+^ cells. Identical gain and gating was employed on all stimulated, unstimulated, pre-, and post-vaccine plots prior to analysis. Representative antigen-stimulated, post-vaccine plots from a IFNγ-responsive patient (#4) are shown

IFNγ and TNFα production was quantified according to “Patients and methods” (Day −7 and Day 56, respectively). TNFα responses were highly correlated with IFNγ responses (*r* = 0.92; *p* = 0.001). Individual patient immune response indexes are presented in Table 4 with representative gating and responder IFNγ plots presented in Fig. 6. For vaccine response determination, post-vaccine values were divided by pre-vaccine values, with a ≥.5-fold increase over pre-vaccine constituting post-vaccine response enhancement. The baseline for the pre-vaccine calculation is cytokine production in the absence of peptide with ≥.5-fold increased production before vaccination constituting and endogenous antigen-directed response as previously published [2, 6]. Antigen-specific cytokine production was attempted by co-staining with immunizing epitope-specific pHLA multimers (NCI), but failed to exhibit convincing pHLA staining in any patient (not shown). Although Day 56 was previously reported to represent the peak response time in similar DC trials [2], immune responsiveness was assessed at later time points in patients with long survival at Day 56. Of these six patients, only one exhibited a previously undetected vaccine response approaching a 1.5-fold increase after Day 56 (1.46-fold; not shown). Analysis of immune responses from samples 7 days before vaccine administration and compared with samples 56 days after the first vaccine showed 5 of 15 GBM patients tested (33 %) exhibited a positive vaccine response of >.5-fold increase. PFS exhibited a trend toward better survival in responders relative to non-responders, as well as increased long-term and overall survivors, but these did not reach significance.

## Discussion

The dendritic cell vaccine evaluated was shown to be non-toxic in this study. Survival analysis of the newly diagnosed patients demonstrating a 16.9-month PFS, together a median overall survival of 38.4 months, suggests that vaccination following conventional treatment may be associated with a clinically relevant response. Enhancement of a type I immune response in 33 % of these patients is comparable to previous DC vaccine trials. Vaccine responders exhibited a non-significant trend toward increased progression-free survival, but not overall survival. Recent clinical trials in glioma patients reported favorable safety and clinical outcomes following DC vaccination [3, 28]. In one trial, a significant correlation between IL-12 produced by DCs in the vaccine with increased time to progression in treated patients was observed [28]. Although no similar correlation of effector T-cell responses and clinical metrics was observed here, as we previously reported [2], the apparent clinical improvement of these patients corroborates that type I cytokine–enhancing DC vaccination may benefit GBM patients.

Our analysis did not reveal significant correlations of vaccine-associated type I cytokine levels within survival metrics, as in previous brain tumor vaccine trials. Although it is possible that with further follow-up some of these metrics may yet reach significance, this continues a predominant trend of poor correlation of immune and survival metrics in cancer vaccine trials. This may be due to inaccurate determination of clinically relevant immune responder status by ex vivo assays, but could also reflect local inhibition of peripherally activated immune effectors at the tumor site. In the case of general assay inaccuracy, long- and short-term survivors are expected to be essentially evenly distributed among responders and non-responders. In the case of local inhibition, however, long-term survivors are expected to be disproportionately represented within immune responders, with short-term survivors essentially evenly distributed, as is evident in our study. Thus, local inhibition may affect the lack of immune-survival correlation more than assay inaccuracy in this study, although it is also possible that our assay may preferentially but imperfectly identify clinically relevant immune responders. One strategy to address this is to identify immune and surrogate biomarkers locally at the tumor site and compare them to both peripheral response status and clinical outcomes. Gene and protein analysis of pre- and post-vaccine gliomas is currently underway to identify candidate biomarkers for this approach.

Evaluation of target tumor antigen expression on patient tumors by PCR showed that all patients expressed at least three of the immunizing antigens, with 74 % of the patients expressing all six. Moreover, quantified expression of AIM-2 and MAGE1 on primary tumors correlated significantly with longer PFS, while expression of AIM-2 and MAGE1 correlated significantly with longer PFS and OS. The level of expression reported here is consistent with a previous study of GBM tumors [19].

Multiple epitopes targeted by our vaccine are derived from proteins reported to be overexpressed on the cancer stem cell fraction of GBM [17]. Moreover, loss of targeted antigen reportedly correlates with glioma vaccine efficacy [29]. Consistent with those findings, we observed a downregulation of three of the HLA-A2 target antigens was observed in four HLA-A2+ patients where recurrent tumors were available. We also examined loss of expression of CD133, a non-targeted antigen expressed on the stem cell fraction in gliomas, to evaluate whether stem-like glioma cells might be selectively targeted by the vaccine. Although only a few patients were studied, tumors from post-vaccine resections showed a decrease in or loss of CD133 expression relative to their pre-vaccine counterparts. This finding is intriguing because previous studies from our group [14] and others [16] have consistently shown increased expression of CD133 in recurrent tumors, including in those recurring after tumor lysate–loaded DC vaccination [30]. Although the relevance of these inconsistent increases to cancer stem and/or endogenous neural stem cells, as well as to clinical disease course, is not entirely clear [14, 16], the observed decreased expression of CD133 is consistent with preferential vaccine-mediated elimination of CD133+ cells from recurrent tumors [17].

In conclusion, this phase I study of ICT-107 demonstrated the feasibility, safety, and bioactivity of a TAA-pulsed dendritic cell vaccine for patients with glioblastoma. Expression of AIM2 and MAGE1 antigens in the pre-vaccine tumors correlated with prolonged survival as measured by PFS and OS in ND-GBM patients, while expression of HER2 and gp100 showed a trend toward longer PFS and OS. With these encouraging results, a randomized, placebo-controlled, phase II trial is underway.
